# Effect of *SIRT1* on white matter neural network in adolescent patients with depression

**DOI:** 10.3389/fpsyt.2022.966315

**Published:** 2022-09-13

**Authors:** Ling Ji, Wen Jiang, Daiyan Liu, Kaiwen Hou

**Affiliations:** ^1^Department of Clinical Psychology, Southwest Hospital, Army Medical University, Chongqing, China; ^2^Outpatient Department, The General Hospital of Western Theater Command, Chengdu, China; ^3^People's Liberation Army of China (PLA) Strategic Support Force Characteristic Medical Center, Beijing, China

**Keywords:** adolescent depression, *SIRT1*, rs12415800, diffusion tensor imaging, white matter

## Abstract

**Background:**

This study aimed to explore the correlation between the rs12415800 polymorphism of the silent information regulator 1 (*SIRT1*) gene and the white matter neural circuit in adolescent patients with depression.

**Methods:**

We enrolled 119 participants, comprising 59 adolescent patients with depression and 60 matched healthy controls for analysis. Patients were further subdivided based on genotype; GG, AG, and AA, with G representing the wild type gene, and A representing the A allele at rs12415800.

**Results:**

We found that: (1) lower anisotropy fraction (FA) values in the left cingulate fasciculus and left anterior thalamus radiation in the AG/AA genotype were more likely to be affected by depression. (2) The FA values of the right inferior occipital-frontal fasciculus, right corticospinal tract, right inferior longitudinal fasciculus, and right superior longitudinal fasciculus regions in the depression (AG/AA) group were lower than in the depression (GG) group. (3) FA values of the right inferior occipital-frontal fasciculus left corticospinal tract, right inferior longitudinal fasciculus, left anterior thalamus radiation, right superior longitudinal fasciculus, left inferior longitudinal fasciculus, left uncinate fasciculus, and right anterior thalamus radiation in the depression (GG) group were lower than the control (GG) group.

**Conclusions:**

The polymorphism locus of the *SIRT1* gene rs12415800 may be related to changes in the microstructure of white matter fiber tracts, and patients carrying the A allele (AG/AA) have more changes in the white matter than those with the non-A allele (GG).

## Introduction

Depression is a common mental disorder affecting more than 264 million people worldwide, and is a leading cause of disability, contributing greatly to the global burden of disease (1). According to previous reports 75% of adult depression patients have a history of depressive episodes in adolescence; adolescent depression has become a high-risk factor for adult depression (2). In addition, it is also a high-risk factor for adolescent suicide, social dysfunction, lack of education, smoking, substance abuse, and obesity (3). It is therefore important to study the mechanisms of adolescent depression.

Previous studies have shown that depression is a complex and heterogeneous brain disease caused by the interaction between genes and the environment (4). Traditional genetic studies, such as family and twin research, have confirmed that depression has a hereditary component, which accounts for ~30–60% of cases (5). In recent years, with the development of genome-wide association studies (GWAS), researchers have found that the *SIRT1* gene rs12415800 locus significantly correlates with depression (6). SIRT1 is a nicotinamide adenine dinucleotide (NAD)-dependent deacetylase belonging to the histone deacetylase (HDAC) family and is a coenzyme-dependent protease that regulates cell function by deacetylating different proteins (7, 8). The *SIRT1* gene, located on the long arm of chromosome 10, is widely expressed in the brain, especially during the development of the hippocampus and cerebral cortex. It participates in a variety of biochemical processes, and exerts a wide range of protective effects on the survival and repair of nerve cells (9).

Animal experiments have suggested that abnormalities in *SIRT1* may be related to the impairment of cognitive function. Rats with a specific *Sirt1* gene knockout or *Sirt1* mutation without catalytic activity develop cognitive impairment, which is related to plasticity-related damage of the hippocampus (10). The symptoms of depression in rats can be significantly improved by increasing the *Sirt1* gene immune response in the hippocampus and hypothalamus following electric shock (11, 12). In the population study, Kishi's study in the Japanese population found an association between *SIRT1* gene and major depressive disorder (MDD) in the allele/genotype analysis (13). Kovanen's study of Finnish population aged 30 years and older also found *SIRT1* Polymorphisms associated with depressive disorders (14). And the results of Tang's study in Han Chinese has suggested that *SIRT1* may be related to MDD (15). CONVERGE team study showed that the OR value of A allele at rs12415800 site of *SIRT1* gene in Chinese adult patients with recurrent depression was >1, suggesting that A allele may be a risk allele for depression (6).

However, how *SIRT1* affects the brain structure of human depression is still unknown, and there are few related studies. Rao's study conducted on healthy participants of the Han Chinese population, found that *SIRT1* gene was significantly associated with gray matter density in two brain cortical regions: the orbital part of the right inferior frontal gyrus and the orbital part of the left inferior frontal gyrus (16). As for the MDD, Liu's study suggested that the rs12415800 single nucleotide polymorphism (SNP) of the *SIRT1* gene is significantly associated with depression, compared with patients lacking the A risk allele, patients with A risk allele exhibited aberrant gray matter volume in the left posterior cerebellar lobe. Besides, in independent Han Chinese postmortem brain and peripheral blood samples, the MDD risk allele of rs12415800 predicted lower *SIRT1* mRNA levels, which was consistent with the reduced expression of this gene in MDD patients compared with healthy subjects (17). Simultaneously, many studies have clearly proposed and confirmed that the anisotropy fraction (FA) value of white matter fiber tracts in patients with depression is decreased, suggesting that patients with depression may have damaged white matter neural network fiber tracts. Deng's study found that MDD showed lower FA in the prefrontal lobe portion of the left anterior thalamic radiation compared to the healthy controls, which was related to negative feelings such as sadness (18). And there were significant reductions in FA in the subgenual and polar stems of the uncinate fasciculus bilaterally, as well as the subgenual and amygdaloid fibers of the cingulum bundle, in depressed patients compared with controls (19). The uncinate fasciculus has three prefrontal stems, the most medial of which extends from BA25 (which deals with mood regulation) and the most lateral of which extends from the dorso-lateral prefrontal cortex (concerned with executive function). Yang's study found that first-episode MDD patients exhibited reduced FA in the left cingulum and the forceps minor, and left-sided white matter tracts abnormalities, which may contribute to the development of anhedonia in depression (20). But those studies didn't explored the relationship between the changes of white matter and genes, the effect of the *SIRT1* gene on the white matter of the brain in patients with depression is still unclear.

Based on previous research conclusions and findings, we hypothesized that *SIRT1* may be involved in the neuropathophysiological mechanisms of adolescent depression by decreasing the FA of the anterior thalamic radiation, the cingulum bundle, and the uncinate fasciculus, influencing the transmission of cognitive, emotional, and executive functions. This study selected adolescent patients with first-episode of untreated depression to explore the relationship between single nucleotide polymorphisms of the *SIRT1* gene rs12415800 and the white matter neural network in adolescent depression.

## Methods

### Subject information

#### Patient enrollment in the depression group

Patient enrollment was performed by psychiatrists from the Department of Clinical Psychology, Southwest Hospital, Army Medical University. The inclusion criteria for patient enrollment were as follows: (1) compliance with the diagnostic criteria for depression according to the Diagnostic and Statistical Manual of Mental Disorders, Volume V (DSM-5); (2) patients experiencing their first episode of depression; (3) patients with a score >20 on the Center for Epidemiological Studies Depression Scale (CES-D); (4) patients with a score >15 on the 9-item Patient Health Questionnaire (PHQ-9); (5) patients aged 12–20 years; (6) patients not treated with any psychotropic drugs or other therapies; (7) presence of no nervous system, major physical, or other mental diseases; and (8) absence of claustrophobia and contraindications for magnetic resonance imaging (MRI).

#### Participant enrollment for the control group

Control enrollment was completed by psychiatrists from the Department of Clinical Psychology, Southwest Hospital, Army Medical University. Healthy controls were recruited from schools and communities, their age, sex and education were matched with the patients in depression group. The CES-D was also used for primary screening. For those with a score ≤ 15, the Structured Clinical Interview for DSM-IV-TR Axis I Disorders Non-patient Edition (SCID-I/NP) and the Schedule for Affective Disorders and Schizophrenia for School-Age Children-Present and Lifetime Version (non-patient part) (K-SADS-PL) were used to evaluate the follow-up screening of participants aged above 18 and under 18 years old, respectively. None had any mental disease, nervous system disease or major physical disease.

Both patients and control participants answered the CES-D and PHQ-9 questionnaires and read and signed the informed consent form reviewed and approved by the Ethics Committee of the Southwest Hospital, Army Medical University. The informed consent was also obtained from the guardians of the participants' after obtaining the participants' consent.

### DNA extraction and genotyping

For DNA extraction, fasting peripheral venous blood samples were collected from each individual and stored in ethylenediaminetetraacetic acid (EDTA) tubes. DNA was extracted from white blood cells using silica-based membrane technology with a genomic DNA extraction kit (TIANGEN DNA extraction kit, DP348-3), according to the manufacturer's recommendations. DNA samples were stored at −80°C until analysis.

Genotyping was performed using a first generation sequencing method. The polymerase chain reaction (PCR) products were detected and purified using PCR amplification, agarose electrophoresis detection, gel recovery, and other steps. The PCR products were sequenced using a 3730XL sequencer (ABI Company, USA). Analysis of the *SIRT1* gene polymorphism rs12415800 was performed by Shanghai Sangon Biotech Co. Ltd.

### MRI acquisition

MRI scanning was conducted on a 3T whole-body MRI machine (MAGNETOM Verio, Siemens Healthcare, Erlangen, Germany), with an 8-channel head array coil at the Southwest Hospital, Army Medical University. Diffusion-weighted images (DTI) data were acquired axially using a spin echo-echo planar imaging (SE-EPI) sequence of 64 diffusion sensitizing directions and eight non-diffusion (B0) volumes, repetition time (TR) = 10,000 ms, echo time (TE) = 92 ms, slice thickness = 2.0 mm, 75 slices, no gap, field of view (FOV) = 230 × 230 mm, acquisiton matrix = 128 × 128, and bval = 1,000 s/mm^2^. Images were acquired following a within-lab standardized process, including cross checks on image acquisition parameters at the time of scanning, with a single MRI technician overseeing all scanning procedures.

### Research grouping

The genotyping results showed that the rs12415800 locus of the *SIRT1* gene contained three genotypes: GG, AG, and AA. Previous studies have shown that the A allele at rs12415800 of the *SIRT1* gene may be a risk allele for depression (6). Therefore, all participants were divided into four groups, according to whether they carried the A risk allele: depression (GG), depression (AG/AA), control (GG), and control (AG/AA) groups.

### Data analysis

SPSS software (version 20.0; IBM Corp, Armonk, NY, USA) was used to analyze general demographic data,; the χ^2^ test was used for enumeration data, *t*-test or one-way analysis of variance (ANOVA) was used for measurement data, and Pearson correlation coefficient was used for the correlation between FA value and duration of disease and the CES-D and PHQ-9 score; *P* < 0.05 was considered statistically significant.

For genotyping data, Hardy–Weinberg analysis was performed to compare the observed and expected frequencies of the rs12415800 polymorphism using the χ^2^ test in all groups using the online analysis software, SHEsis (http://analysis.bio-x.cn) (21), with *P* > 0.05 indicating that the Hardy–Weinberg equilibrium was met. The distribution of the *SIRT1* rs12415800 SNP in the depression and control groups was compared by analyzing the genotype and allele frequency. Differences were considered statistically significant at *P* < 0.05.

The Digital Imaging and Communication in Medicine (DICOM) format data was converted to the Net InFormation Transfer Index (NIfTI) format, and the DTI data was analyzed using the FMRIB Software Library (FSL) (https://fsl.fmrib.ox.ac.us/fsl/fslwiki) (22) for data processing. Preprocessing followed the standard pipeline, including EPI distortion correction, motion and eddy current distortion correction, skull-stripping with the brain extraction tool, and diffusion tensor model fitting. Output included separate image for FA. The FA image was then nonlinearly registered to a standard template (FMRIB58) followed by affine-alignment to Montreal Neurological Institute standard space (MNI, 1 × 1 × 1 mm) using Tract-Based Spatial Statistics (TBSS) (23, 24). A study-specific, averaged whole-brain skeletonized FA mask was created through TBSS (threshold = 0.2).

The general linear model (GLM) function was then used to perform a 2 × 2 two-way ANOVA design. The two factors were diagnosis and genotype, with two levels for each factor. The diagnosis was classified as depression and normal, and the genotype was classified as genotype GG or AG/AA. At the same time, age, sex, and education level were considered as covariables. Permutation-based non-parametric testing (5,000 permutations) was performed using a 2 × 2 two-way ANOVA analysis. The threshold-free cluster enhancement (TFCE) method was used to perform multiple comparative correction on the FA value results, and *P* < 0.05 was considered statistically significant. The FA values of these areas were then extracted and submitted to *post-hoc* analysis, testing the homogeneity of variances by the Levene's test, and using the Bonferroni method; *P* < 0.05 was considered statistically significant.

## Results

### General characteristics of the participants

The depression group consisted of 59 patients with depression (11 boys and 48 girls), with an average age of 17.78 ± 3.45 years. The control group consisted of 60 participants (nine boys and 51 girls), with an average age of 17.58 ± 1.23 years. There were no significant differences in sex, age, or years of education between the patient and control groups (*P* > 0.05); however, there were significant differences in the CES-D and PHQ-9 scores between the depression and control groups (*P* < 0.05). There were no significant differences in sex, age, or years of education between the depression (GG), depression (AG/AA), control (GG), and control (AG/AA) groups (*P* > 0.05). The demographic characteristics of the participants are shown in Table 1.

**Table 1:** Descriptive characteristics and genotyping data of the depression and control groups.

	**Age**	**Education year**	**Sex (male/female)**	**CES-D**	**PHQ-9**	**Duration of disease (month**,	**Allele frequency**	**Genotype frequency**	**Hardy–Weinberg** **equilibrium**	
	**(*x* ±s)**	**(*x* ±s)**		**(*x* ±s)**	**(*x* ±s)**	***x* ±s)**	**A/G**	**AA/AG/GG**	**χ^2^**	***P-*value**
Depression group (*n* = 59)	17.78 ± 3.45	11.84 ± 2.34	11/48	42.83 ± 6.87	19.08 ± 4.30	6.70 ± 3.13	52/66	11/30/18	0.058	0.808
							0.441/0.559	0.186/0.509/0.305		
Control group (*n* = 60)	17.58 ± 1.23	12.48 ± 1.56	9/51	8.88 ± 5.69	2.55 ± 2.50		61/59	18/25/17	1.662	0.197
							0.508/0.492	0.300/0.417/0.283		
t/χ^2^	−0.411	1.463	0.283	−29.355	−25.568		1.092	2.164		
*P-*value	0.682	0.147	0.595	0.000	0.000		0.295	0.338		
Depression (GG) group (*n* = 18)	16.61 ± 2.14	11.30 ± 1.99	3/15	43.72 ± 6.75	19.83 ± 4.03	5.50 ± 3.14				
Depression (AG/AA) group (*n* = 41)	18.29 ± 3.80	12.10 ± 2.47	8/33	42.44 ± 6.97	18.76 ± 4.43	7.15 ± 3.03				
Control (GG) group (*n* = 17)	18.06 ± 1.48	12.76 ± 1.64	4/13	8.76 ± 4.96	2.41 ± 2.24					
Control (AG/AA) group (*n* = 43)	17.40 ± 1.09	12.23 ± 1.52	5/38	8.93 ± 6.02	2.6 ± 2.62					
t*/F*/χ^2^	2.158	1.730	1.589	283.786	218.655	−2.16				
*P-*value	0.097	0.165	0.662	0.000	0.000	0.034				

### Genotyping of *SIRT1* rs12415800

Hardy–Weinberg analysis revealed that both the depression group (χ^2^ = 0.058, *P* = 0.808) and control group (χ^2^ = 1.662, *P* = 0.197) met Hardy–Weinberg equilibrium (*P* > 0.05). There were no statistically significant differences in genotype frequency or allele frequency between the two groups (*P* > 0.05), as shown in Table 1.

### Genotype and diagnostic interaction

**Figure 1:**
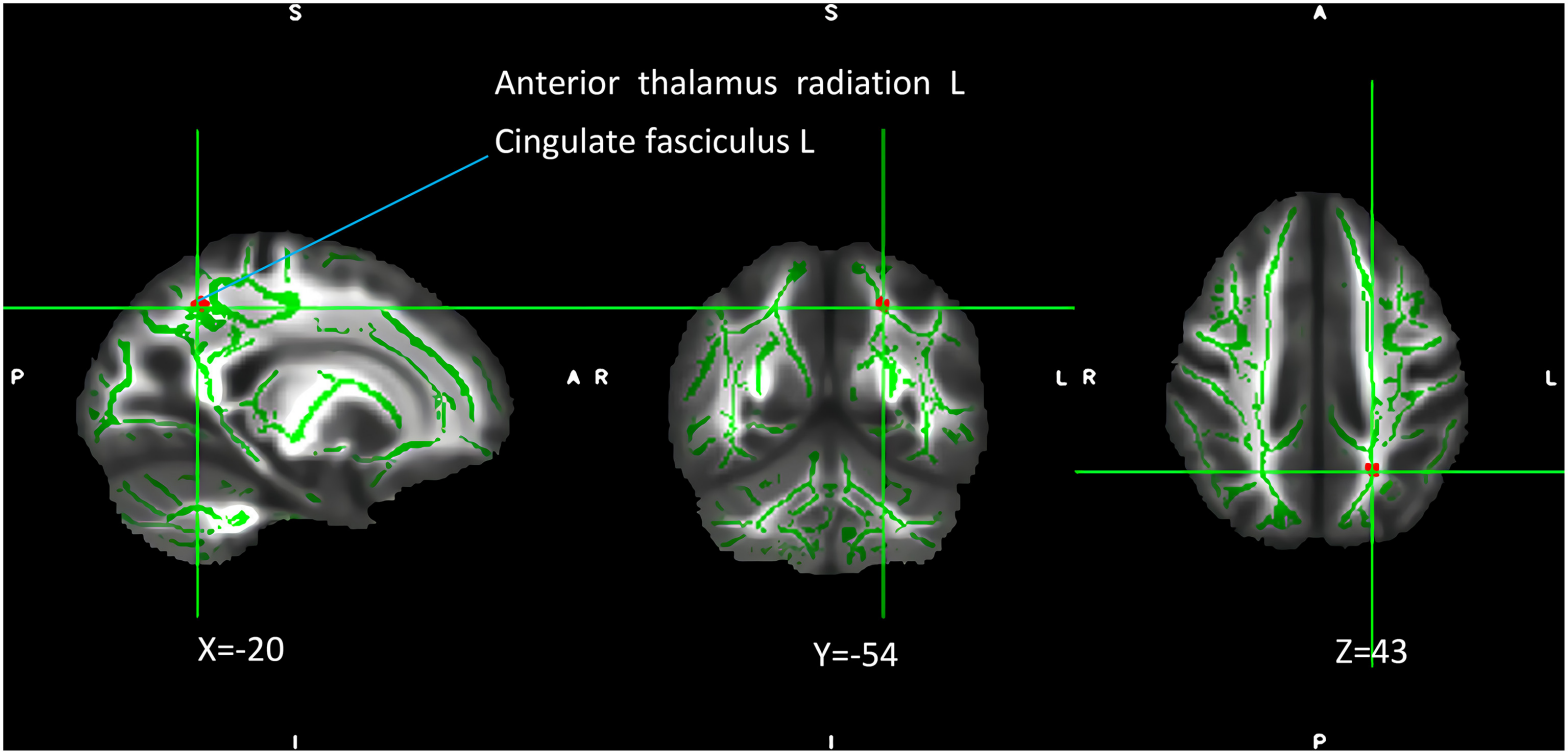
Interaction of genotype and diagnosis on FA among the four groups. The green regions indicate FA white matter skeletons. Red-yellow regions indicate significantly different FA values between the four groups. All statistical maps were thresholded at *P* < 0.05 (after TFCE correction for multiple comparisons).

**Figure 2:**
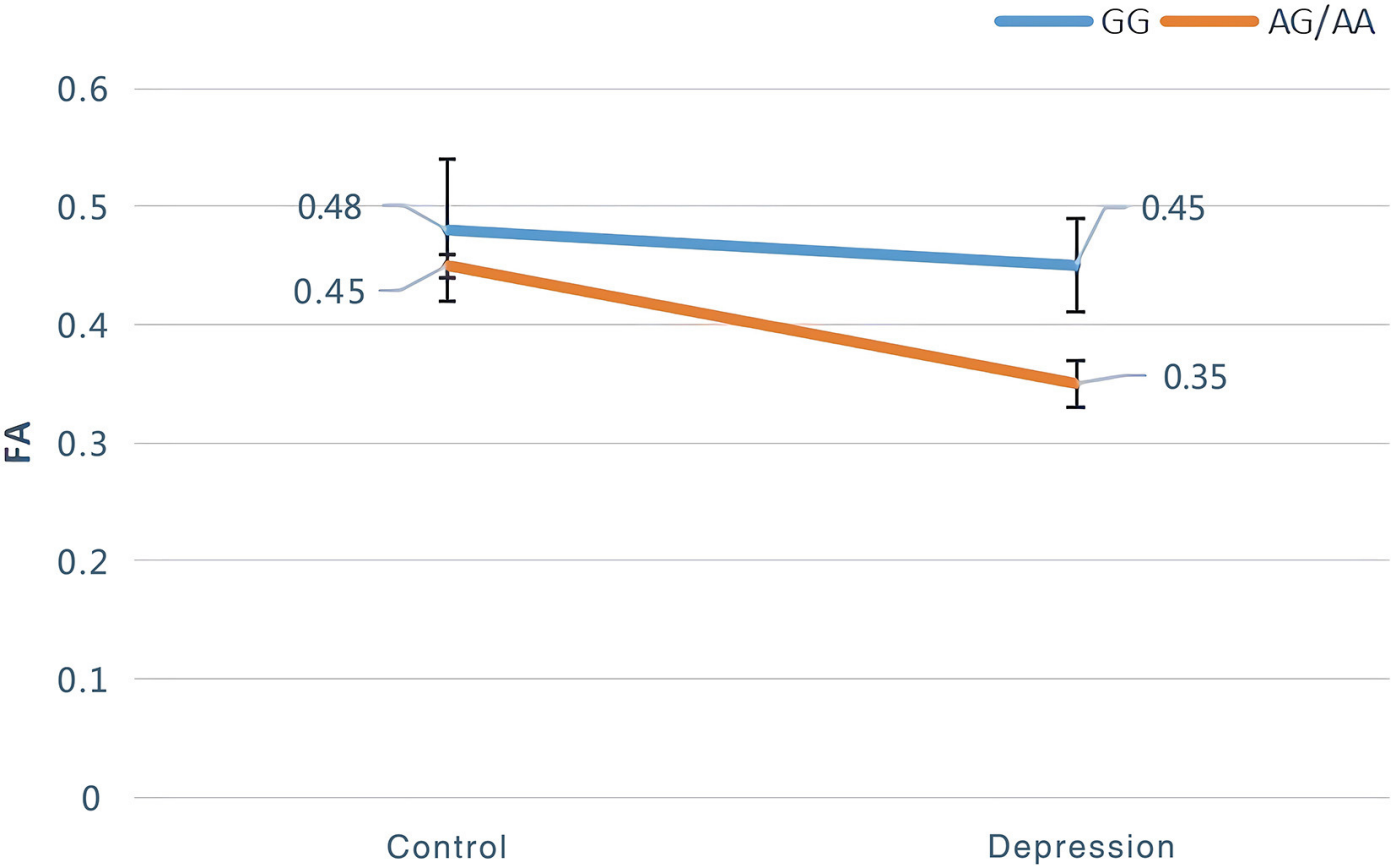
*Post-hoc* analysis of genotype and diagnostic interaction of FA among the four groups.

The interaction of the *SIRT1* gene rs12415800 genotype and diagnosis of FA values in the depression (GG), depression (AG/AA), control (GG), and control (AG/AA) groups showed that there was a significant difference in one cluster, which contained the left anterior thalamus radiation (2.0%) and left cingulate fasciculus (1.3%) (MNI coordinates *X* = −20, *Y* = −54, *Z* = 43; voxel = 36; *P* = 0.032) (Figure 1). The *post-hoc* analysis revealed that the FA value of depression (AG/AA) group was the lowest. Both in the control group and depression group, when the genotype was AG/AA, the FA value was lower than that of genotype GG, the average FA value of control group (AG/AA) was 0.03 lower than that of the control group (GG) (*P* < 0.001), the average FA value of depression group (AG/AA) was 0.1 lower than that of the depression group (GG) (*P* < 0.001). With genotype GG, the average FA value of depression (GG) group was 0.03 lower than that of the control group (GG) (*P* < 0.001); with the genotype AG/AA, the average FA value of depression (AG/AA) group was 0.1 lower than that of the control group (AG/AA) (*P* < 0.001), and when the genotype was AG/AA, compared with the genotype GG, the average FA value of the depression group decreased more than that of the control group (Figure 2).

### Main effect of genotype

**Figure 3:**
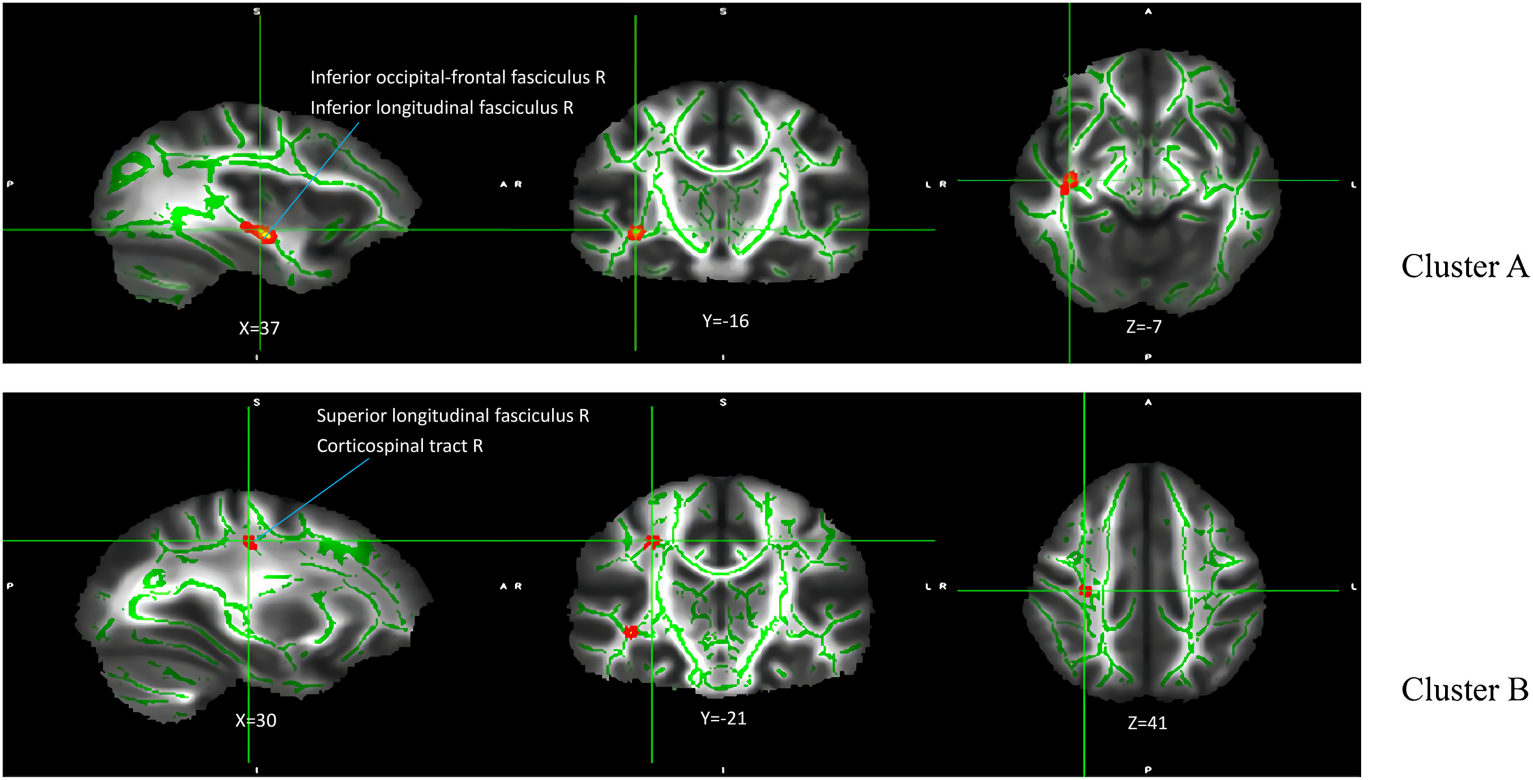
Main effect of genotype on FA among the four groups. The green regions indicate FA white matter skeletons. Red-yellow regions indicate significantly different FA between the four groups. All statistical maps are thresholded at *P* < 0.05 (after TFCE correction for multiple comparisons).

**Figure 4:**
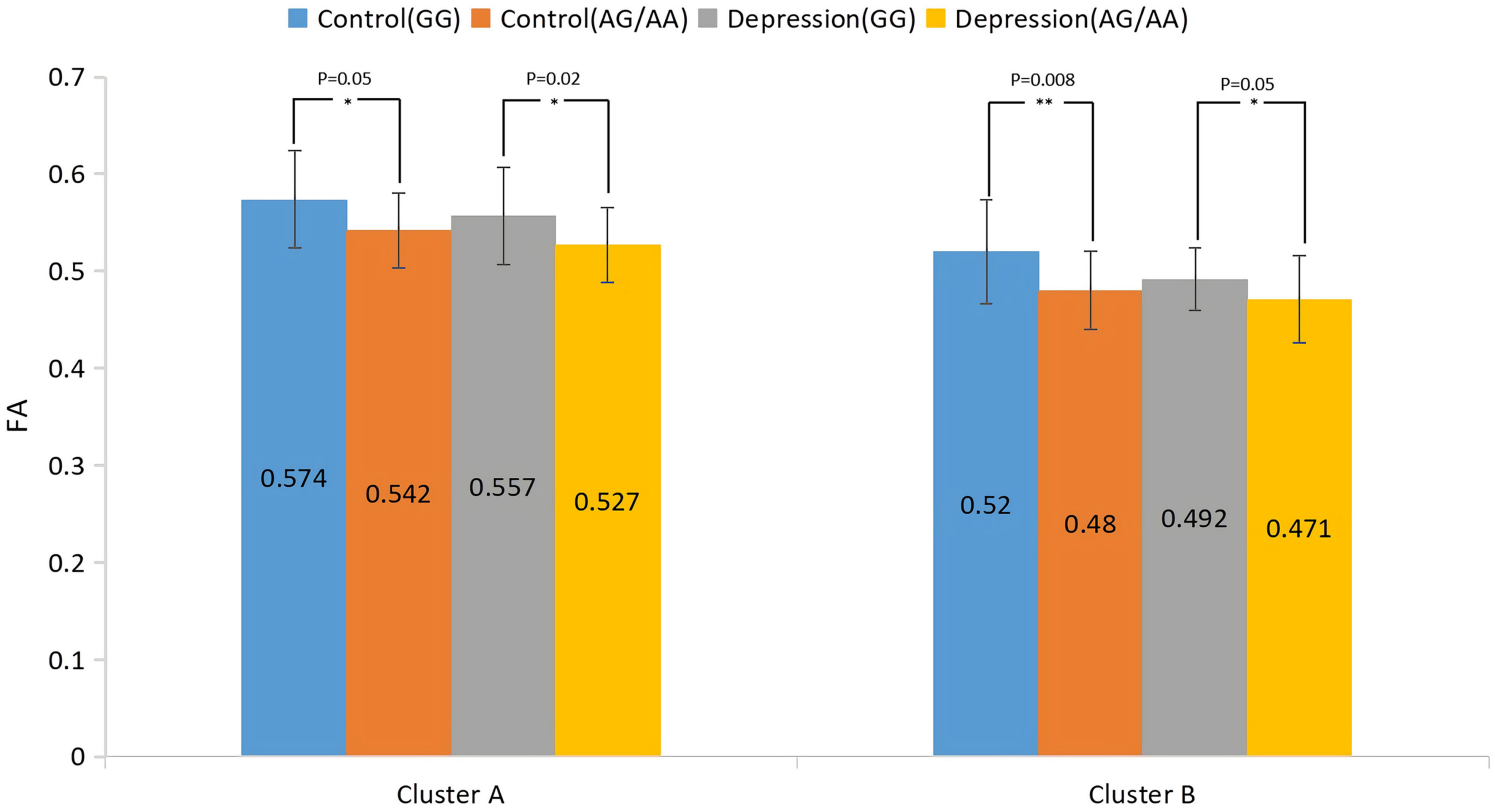
*Post-hoc* analysis of the main effect of genotype on FA among the four groups. *P < 0.05, **P < 0.01.

The results of the analysis of the main effect of genotype on FA values among the depression (GG), depression (AG/AA), control (GG), and control (AG/AA) groups showed that there were significant differences between the two clusters, including the right inferior occipital-frontal, right inferior longitudinal, and right superior longitudinal fasciculus and right corticospinal tract (Table 2; Figure 3). *Post-hoc* analysis revealed that the average FA value of the control (AG/AA) group was lower than that of the control (GG) group, and the average FA value of the depression (AG/AA) group was lower than that of the depression (GG) group (Figure 4).

**Table 2:** Main effect of genotype on FA among the four groups.

**Cluster**	**White matter fiber bundles**	**Voxel**	**MNI coordinates**			***P*-value**
			***X***	***Y***	***Z***	
Cluster A	Right inferior occipital-frontal fasciculus (34.7%)	8	37	−16	−7	0.022
	Right inferior longitudinal fasciculus (6.0%)					
Cluster B	Right superior longitudinal fasciculus (26.7%)	66	30	−21	41	0.018
	Right corticospinal tract (1.7%)					

### Main effect of diagnosis

**Figure 5:**
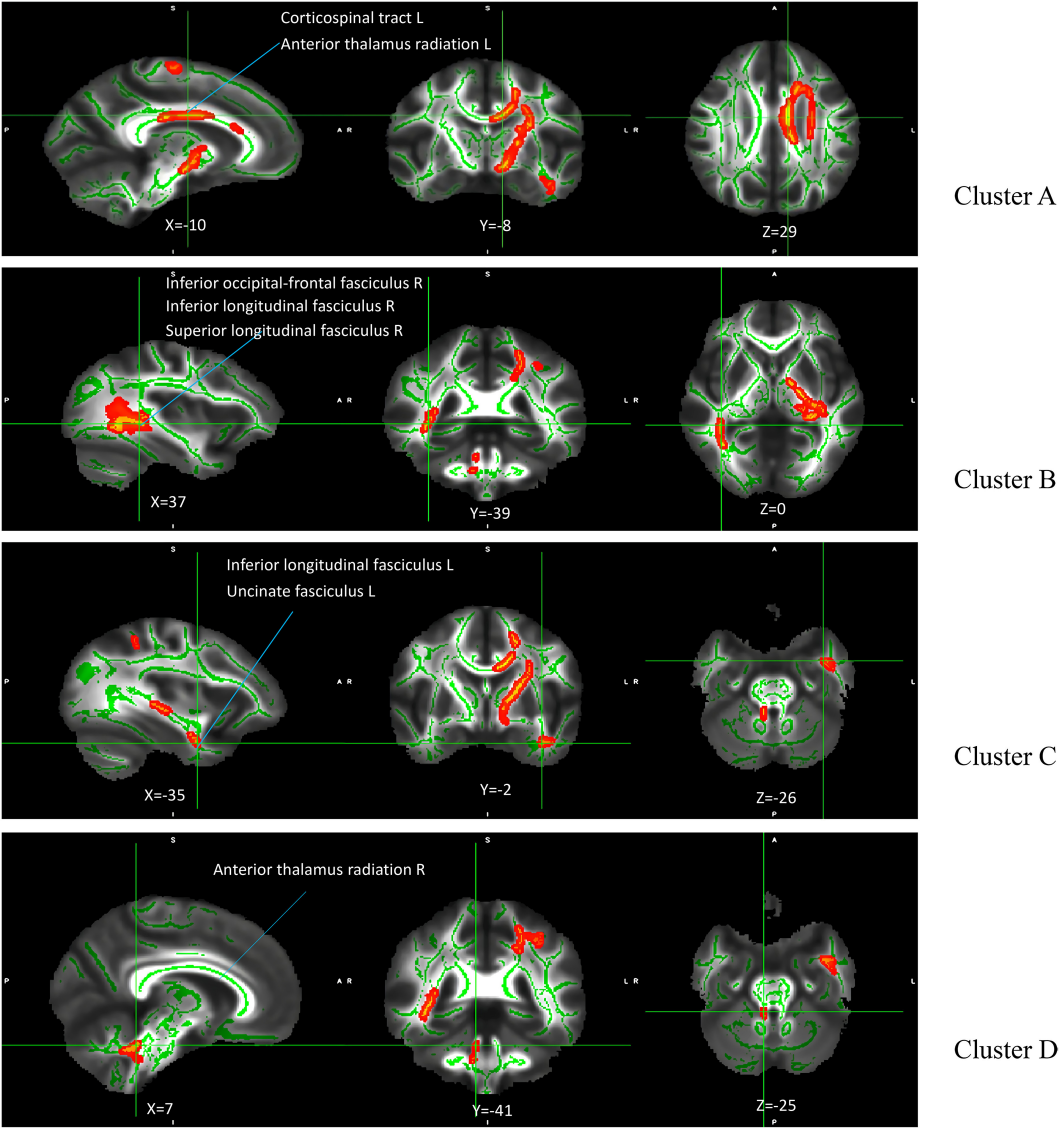
Main effect of diagnosis on FA among the four groups. The green regions indicate FA white matter skeletons. Red-yellow regions indicate significantly different FA between the four groups. All statistical maps are thresholded at *P* < 0.05 (after TFCE correction for multiple comparisons).

**Figure 6:**
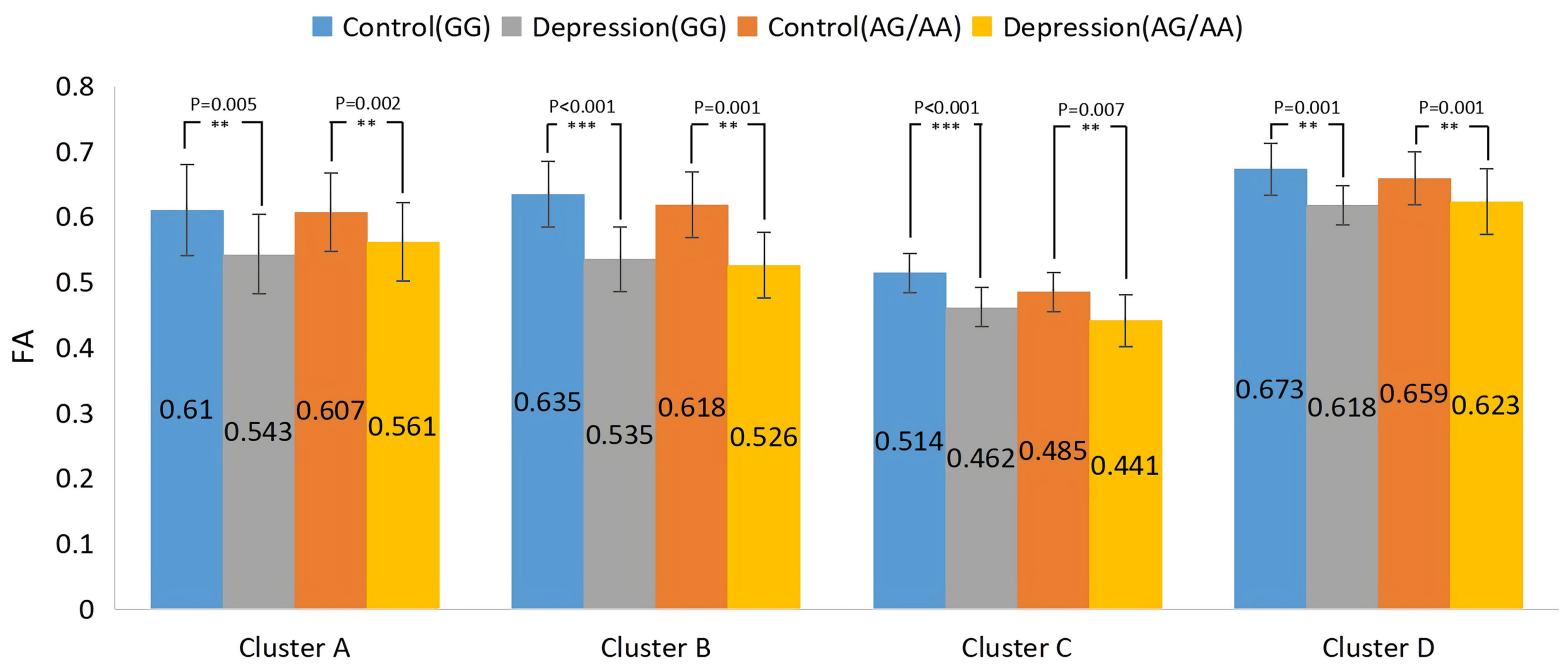
*Post-hoc* analysis of the main effect of diagnosis on FA among the four groups. **P < 0.01, ***P < 0.001.

The results of the main effect of diagnosis on FA values among the depression (GG), depression (AG/AA), control (GG), and control (AG/AA) groups showed that there were significant differences in four clusters, including the left corticospinal tract, left and right anterior thalamus radiation and right inferior occipital-frontal, right inferior longitudinal, right superior longitudinal, left inferior longitudinal, and left uncinate fasciculus (Table 3; Figure 5). *Post-hoc* analysis revealed that the average FA value of the depression (GG) group was lower than that of the control (GG) group, and the average FA value of the depression (AG/AA) group was lower than that of the control (AG/AA) group (Figure 6).

**Table 3:** Main effects of diagnosis on FA among the four groups.

**Cluster**	**Bundles of white matter fibers**	**Voxel**	**MNI coordinates**			***P*-value**
			***X***	***Y***	***Z***	
Cluster A	Left corticospinal tract (7.1%)	4,221	−10	−8	29	0.002
	Left anterior thalamus radiation (3.4%)					
Cluster B	Right inferior occipital-frontal fasciculus (25.3%)	517	37	−39	0	0.011
	Right inferior longitudinal fasciculus (16.1%)					
	Right superior longitudinal fasciculus (1.8%)					
Cluster C	Left inferior longitudinal fasciculus (16.6%)	142	−35	−2	−26	0.029
	Left uncinate fasciculus (6.3%)					
Cluster D	Right anterior thalamus radiation (6.8%)	79	7	−41	−25	0.026

### Clinical index and scale analysis

There was no statistical difference between CES-D and PHQ-9 score between the depression (GG) and depression (AG/AA) groups (*t* = 0.657, *P* = 0.514; *t* = 0.883, *P* = 0.381), but there were statistical differences between the duration of disease (*t* = −2.16, *P* = 0.034), and the duration of depression (AG/AA) group was longer (Table 1).

## Discussion

To the best of our knowledge, this is the first study to explore the relationship between the *SIRT1* gene rs12415800 single nucleotide polymorphism and the white matter neural network in adolescent depression patients. These results suggest that there is an interaction between diagnosis and genotype in adolescent depression. The obvious interaction areas included the left anterior thalamus radiation and left cingulate fasciculus. The depression (AG/AA) group had the lowest FA values. Compared with genotype GG, when the genotype was AG/AA, the depression group had a greater decrease in FA value than that of the control group. We further found that genotype and diagnosis have their own main effects. In terms of the main effects of genotype, the four groups had statistical differences in FA values in the, right corticospinal tract and right inferior occipital-frontal right inferior longitudinal, and right superior longitudinal fasciculus. For groups with the same diagnosis, the average FA value in the AG/AA genotype group was lower than that in the GG genotype group. In terms of the main effect of diagnosis, the four groups had statistical differences in FA values in the left corticospinal tract, left and right anterior thalamus radiation, and right inferior occipital-frontal, right inferior longitudinal, right superior longitudinal, left inferior longitudinal, and left uncinate fasciculus, and. In the above area, the FA value of the depression group was lower than that of the control group when they had the same genotype.

The *SIRT1* gene is widely involved in the development of the brain morphology and is related to neural development. The early growth of individual nerves, including axon growth, subsequent axon differentiation, dendritic structure, and synapse formation, are all related to the function of *SIRT1* (25). *SIRT1* further regulates the production of cAMP response element binding and brain-derived neurotrophic factor by regulating microRNA-138 (26), both of which have been found to be associated with depression (27). Our research results are consistent with those of the CONVERGE team, which found that the *SIRT1* gene is related to depression in the Han population (6). Regarding the areas affected by the interaction effect, both diagnostic and genotypic factors have an impact on these areas, and the effect of one factor changes with a change in the other factor. The anterior thalamus radiation connecting the frontal lobe and thalamus participates in the transmission of neural information in the subfrontal cortex circuit and can receive and transmit neural signals related to executive function (28). Moreover, it is related to negative affective regulation and interacts with the medial forebrain tract to jointly regulate the affective response system (29). Furthermore, combined with upper longitudinal tract fibers, the anterior thalamus radiation may also be related to cognition (30). Damage to its microstructure may affect emotional, cognitive, and executive functions. The left cingulate fasciculus is an important part of the reward loop, and is related to higher cortical functions, such as motivation, associative learning, and positive emotional feelings (31). Damage to the fiber bundles may impair the normal function of the reward loop such that past happy experiences cannot enter the reward pathway, resulting in the same things failing to stimulate pleasant feelings, resulting in symptoms such as anhedonia and depression (32). Previous studies have observed a decrease in FA values in the above areas in adolescents, middle-term youth, and elderly people with depression (33, 34), suggesting that damage to these white matter areas is contributes to the pathophysiological process of depression. In our study, both controls and patients with depression carrying the A allele of the *SIRT1* gene rs12415800 exhibited more severe damage to the white matter structure of the adolescent brain. This indicates that different genotypes of the *SIRT1* gene rs12415800 affect the left cingulate fasciculus and left anterior thalamus radiation, causing changes in the microstructure of the white matter fiber bundles of these regions, potentially influencing cognitive, executive, and emotional functions.

Both the genotype and diagnosis had significant main effects in the right inferior occipital-frontal fasciculus, right inferior longitudinal fasciculus, and right superior longitudinal fasciculus. The inferior occipital-frontal fasciculus connects the dorsolateral prefrontal lobe, parietal lobe, temporal lobe, posterior occipital lobe, and cerebral cortex of the cingulate gyrus, and participates in the processes of thinking, awakening, and executive and cognitive functions (35). Many studies on depression have found that the FA value of the inferior occipital-frontal fasciculus in patients with depression is lower (36) and correlates with higher serum levels of interleukin (IL)-1β in, suggesting that inflammatory factors may be involved in the process of its microstructural damage (37). Several studies have further shown that the *SIRT1* gene can participate in neuro-immuno-inflammatory regulation by regulating IL-1β (38). Therefore, the *SIRT1* gene may change the microstructure of the subfrontal tract by regulating the immune-inflammatory mechanism, thereby affecting visual, emotional, and executive functions of patients with depression. In addition, the inferior occipital-frontal fasciculus and inferior longitudinal fasciculus connect the visual cortex and emotion-related brain areas and participate in visual emotion functions (39). The inferior longitudinal fasciculus is also involved in emotional visual function and visual memory, which are related to emotional disorders (40). The superior longitudinal fasciculus participates in the function of the “frontal cortex-marginal-striatum-globus pallidus-thalamus” circuit, which functions to maintain emotional stability and response to emotional stimuli and affects the body's emotional regulation function (41). This circuit is also involved in advanced cortical functions, such as cognition and executive function (42). Damage to the white matter microstructure in the above areas may lead to abnormalities in mood and cognitive and executive function associated with depression. Regardless of whether the diagnosis factor was depression or normal, adolescents carrying the A allele of the *SIRT1* gene rs12415800 may have more white matter microstructure damage in the above regions.

More affected regions were found regarding the main effect of diagnosis, including the left corticospinal tract, left inferior longitudinal fasciculus, left uncinate fasciculus, and right anterior thalamus radiation. A possible to explanation for this is that the pathological mechanism of depression involves the participation of multiple genes and environmental factors (43), and thus, the contribution of a single gene is limited to identify all significant results. The corticospinal tract connects the white matter fiber bundles in brain regions, such as the primary motor, premotor, and somatosensory cortex, parietal lobe, auxiliary motor area, and cingulate gyrus (44). When this connection is abnormal, the information transmission of the cortex and subcortical brain area is impaired, showing “disconnection syndrome,” leading to psychomotor retardation (45). In addition, it is anatomically connected to the somatosensory cortex, cingulate cortex, and insular cortex, which are related to emotional and cognitive functions. The change in its microstructure is related to a decrease in perception speed (46). When faced with a fearful situation, the corticospinal tract may be involved in the relevant emotional reaction process (47). Therefore, any damage may affect cognitive ability and the emotional response process and may play a role in the pathophysiological process of psychomotor retardation in depression. The uncinate fasciculus connects the orbital frontal cortex, hippocampus, and amygdala in the frontal limbic system and plays an important role in emotion and cognitive functions (48). Damage to its microstructure may affect contextual memory (49) and emotional social ability (50). Previous studies have shown that the FA value of the uncinate fasciculus in patients with depression is reduced (19).

The duration of disease in the depression (AG/AA) group was longer than that in the depression (GG) group, but there was no difference in CES-D and PHQ-9 score between the two groups. As an important indicator of disease, the duration of disease reflects the duration of disease damage to individuals; importantly, we found that the duration of disease was longer in the depression (AG/AA) group, which indirectly reflects that adolescent depression patients with *SIRT1* gene rs12415800 locus A allele may experience longer damage to the brain white matter microstructure than those not carrying A allele. The possible reason is that CES-D and PHQ-9 are a self-report scale, and each person has different feelings and understanding of the severity of the symptoms, which is a subjective factor in the assessment process, while the duration of the disease is relatively more objective. This fact may also be related to the limited items of the scales and the small sample size.

Nevertheless, the present study has several limitations. First, due to the limited number of samples, the analysis of the AG and AA genotypes at the rs12415800 locus of the *SIRT1* gene was combined and could not be analyzed separately, which limits the results. Thus, these conclusions need to be confirmed in a larger sample. Moreover, this was a cross-sectional study. Although we found that the *SIRT1* gene rs12415800 single nucleotide polymorphism is associated with adolescent depression, it is impossible to conduct further mechanistic explorations. Thus, prospective studies are essential to verify our conclusions. Depression is a disease involving multiple genes and environmental and epigenetic factors. Exploring the influence of nucleotide polymorphisms at a single locus of a single gene on depression leads to limited results. Subsequent multi-gene and multi-factor studies are needed to reveal additional findings.

## Conclusions

This study found that there is damage to the microstructure of the white matter neural network fiber bundles in adolescent patients with depression, in which the genetic polymorphism of the *SIRT1* gene rs12415800 locus plays an important role. Carrying the A allele may result in greater damage to the white matter neural network in both healthy and depressed adolescents. Furthermore, microstructural damage may lead to changes or disturbances in the function of the white matter neural network, which may eventually affect cognitive and emotional regulation functions in adolescent patients with depression.

## Data availability statement

The raw data supporting the conclusions of this article will be made available by the authors, without undue reservation.

## Ethics statement

The studies involving human participants were reviewed and approved by Southwest Hospital, Army Medical University. Written informed consent to participate in this study was provided by the participants' legal guardian/next of kin. Written informed consent was obtained from the individual(s), and minor(s)' legal guardian/next of kin, for the publication of any potentially identifiable images or data included in this article.

## Author contributions

LJ, WJ, and KH conceived and designed the experiments, analyzed the data, and wrote the paper. DL performed the experiments and analyzed the data. All authors have read and approved the final version of this manuscript.

## Conflict of interest

The authors declare that the research was conducted in the absence of any commercial or financial relationships that could be construed as a potential conflict of interest.

## Publisher's note

All claims expressed in this article are solely those of the authors and do not necessarily represent those of their affiliated organizations, or those of the publisher, the editors and the reviewers. Any product that may be evaluated in this article, or claim that may be made by its manufacturer, is not guaranteed or endorsed by the publisher.
